# Editorial: Omics in endocrinology: from biomarker discovery to targeting therapeutic strategies

**DOI:** 10.3389/fmolb.2025.1567250

**Published:** 2025-02-13

**Authors:** Michele Costanzo, Shereen M. Aleidi, Anas Abdel Rahman

**Affiliations:** ^1^ Department of Molecular Medicine and Medical Biotechnology, University of Naples Federico II, Naples, Italy; ^2^ CEINGE–Biotecnologie Avanzate Franco Salvatore, Naples, Italy; ^3^ Department of Medicinal Chemistry, College of Pharmacy, University of Sharjah, Sharjah, United Arab Emirates; ^4^ Department of Biopharmaceutics and Clinical Pharmacy, School of Pharmacy, The University of Jordan, Amman, Jordan; ^5^ Metabolomics Section, Department of Clinical Genomics, Genomics Medicine Center of Excellence, King Faisal Specialist Hospital and Research Centre (KFSHRC), Riyadh, Saudi Arabia; ^6^ Department of Biochemistry and Molecular Medicine, College of Medicine, Alfaisal University, Riyadh, Saudi Arabia

**Keywords:** metabolic disease, metabolomics, precision medicine, endocrinolgy, diabetes mellitus, thyroid disease

Type 2 diabetes mellitus (T2DM) and thyroid disease (TD) are two endocrine disorders that are closely linked to metabolic dysfunction, thus representing established health concerns worldwide (Kalra et al., 2019). T2DM is a chronic condition marked by β-cell dysfunction, abnormal glucose homeostasis, and insulin resistance (DeFronzo et al., 2015). Thyroid diseases involve thyroid hormone production and regulation abnormalities, affecting glucose metabolism and the immune response (Chauhan and Patel, 2024; Yamauchi and Yabe, 2025). Both conditions significantly influence the quality of life of patients and increase their risk of developing other health complications, such as cardiovascular disease, kidney failure, and neurological impairments. Furthermore, obesity is associated with the incidence of these metabolic diseases (Pulgaron and Delamater, 2014). The key mechanisms underlying these metabolic disorders are complex and multifactorial. In diabetes, the dysfunction in pancreatic β-cells leads to inadequate insulin production, inducing loss of insulin sensitivity in peripheral tissues, such as muscle and adipose tissue (Donath and Shoelson, 2011). Moreover, systemic chronic inflammation, dysregulated adipokine secretion, and altered lipid metabolism relate to the progression of T2DM (Gasmi et al., 2021; Chen et al., 2022). Genetic and environmental factors in TD contribute to altered thyroid function, frequently driven by autoimmune mechanisms (Tomer and Davies, 2003; Bao et al., 2021).

With well-established guidelines to diagnose such disorders, the development of new classes of medications continues (McGill et al., 2024; Tywanek et al., 2024), whereas nanotechnology may revolutionize endocrine disorder treatments, bridging diagnostics and therapies (Yang et al., 2013; Yan et al., 2024). Nonetheless, omics technologies have significantly advanced the discovery of metabolic disorders (Costanzo et al., 2024), for monitoring biomarkers of disease progression, predicting treatment response, and improving follow-up care in diabetes and thyroid disorders. These approaches may enable diagnosis that is more precise and personalized treatment strategies (Olivier et al., 2019; Beger et al., 2020). Metabolomics, in particular, may lean on mass spectrometry (MS) or nuclear magnetic resonance (NMR) strategies to identify, characterize, and monitor molecules for clinical purposes (Roviello et al., 2014; Costanzo et al., 2022; Aleidi et al., 2023; Costanzo and Caterino, 2023). Further multi-omics data integration through advanced computational models has been proven instrumental in validating and prioritizing disease biomarkers for clinical use (Pietzner et al., 2018; Reel et al., 2021; Zhang et al., 2024).

This Research Topic collected original research articles that provide advances through multi-omics strategies to many aspects of endocrinological and metabolic diseases, including thyroid disease, diabetes, and obesity. Given the influence of thyroid hormones on lipids, insulin secretion, and carbohydrate metabolism, their involvement in developing T2DM is expected. To this purpose, using joint models of longitudinal and time-to-event data, Amirabadizadeh et al. investigated the association between serum changes of thyroid-stimulating hormone (TSH) and free thyroxine (FT4) levels and the incidence of T2DM [19]. They used the data from 1938 individuals in the Tehran Thyroid Study cohort and identified new cases of T2DM. Their findings revealed some dynamic variations in serum thyroid hormones connected with the development of T2DM. A significant reverse association between serum TSH levels and the risk of T2DM was demonstrated. These results suggested an intricate interplay between thyroid activity and diabetes risk, highlighting the significance of screening thyroid hormones as a preventive approach for T2DM.

Aleidi et al. investigated the metabolic profiles connected with Gestational Diabetes Mellitus (GDM) using an MS-based untargeted metabolomics approach [20]. Based on the oral glucose tolerance test (OGTT), 40 pregnant women at 24–28 weeks of gestation were screened, and 20 of them were identified as affected by GDM, while the other 20 were used as control. The analysis revealed distinctive metabolic differences between GDM and healthy women, with a significant number of dysregulated metabolites in serum from GDM ones. The most relevant metabolic pathways associated with the altered GDM metabolome included tryptophan metabolism, inositol phosphate metabolism, phenylalanine metabolism, and histidine metabolism. Moreover, the authors selected a set of 10 metabolites, including N-acetylproline and serylmethionine, whose combination showed high diagnostic importance with a high AUC value (0.978). Importantly, this set of metabolites could potentially be tested as novel biomarkers detectable with high specificity in place of standard OGTT to diagnose GDM.

Masood et al. explored the plasma proteome changes following the treatment with liraglutide, a long-acting agonist of GLP-1 receptor, showing strong therapeutic interest worldwide for its role in weight loss and obesity treatment. GLP-1 signaling is proposed to be activated by receptor binding and activation of the cAMP cascade, resulting in effective glucose regulation, improved insulin secretion, decreased glucagon secretion, lower food intake, and increased satiety. However, to elucidate the molecular mechanisms connected with liraglutide effects, the authors applied a label-free quantitative proteomics approach to identify altered pathways and potential biomarkers detectable in plasma from patients before initiating treatment and after 3 months of 3 mg liraglutide therapy. Proteome changes included a reduction of inflammation and oxidative stress pathways and a boost of glycolytic and lipolytic metabolic pathways.

Finally, Tang et al. investigated the metabolic changes and relative pathways associated with exposure to different doses of hexavalent chromium Cr(VI), being established its involvement in carbohydrate, lipid, and nucleic acid metabolism, as well as in the association with diseases such as cardiovascular diseases, diabetes, and depression. They studied the potential neuro-health risks associated with Cr(VI), analyzing the metabolome of rat astrocytes via untargeted metabolomics. Specifically, they highlighted the critical roles of sphingolipid metabolism and the methionine-cysteine cycle in neurotoxicity, with sphingolipid metabolism associated with apoptosis and the methionine-cysteine cycle playing a significant role in oxidative damage.
